# Investigation of the Flow Properties of CBM Based on Stochastic Fracture Network Modeling

**DOI:** 10.3390/ma12152387

**Published:** 2019-07-26

**Authors:** Bo Zhang, Yong Li, Nicholas Fantuzzi, Yuan Zhao, Yan-Bao Liu, Bo Peng, Jie Chen

**Affiliations:** 1 State Key Laboratory of Coal Mine Disaster Dynamics and Control, Chongqing University, Chongqing 400044, China; 2 School of Resources & Safety Engineering, Chongqing University, Chongqing 400044, China; 3 Technology Center of Sichuan Coal Industry Group, Chengdu 610091, China; 4 DICAM, University of Bologna, 40126 Bologna, Italy; 5 Sinohydro Bureau 8 Co. LTD., POWERCHINA, Changsha 410004, China; 6 CCTEG Chongqing Engineering Co. LTD., Chongqing 400016, China

**Keywords:** Monte Carlo method, coalbed methane, stochastic fracture network, fracture geometric parameters, dual-porosity and dual-permeability media, finite element method

## Abstract

Coal contains a large number of fractures, whose characteristics are difficult to describe in detail, while their spatial distribution patterns may follow some macroscopic statistical laws. In this paper, several fracture geometric parameters (FGPs) were used to describe a fracture, and the coal seam was represented by a two-dimensional stochastic fracture network (SFN) which was generated and processed through a series of methods in MATLAB. Then, the processed SFN image was able to be imported into COMSOL Multiphysics and converted to a computational domain through the image function. In this way, the influences of different FGPs and their distribution patterns on the permeability of the coal seam were studied, and a finite element model to investigate gas flow properties in the coal seam was carried out. The results show that the permeability of the coal seam increased with the rising of fracture density, length, aperture, and with the decrease of the angle between the fracture orientation and the gas pressure gradient. It has also been found that large-sized fractures have a more significant contribution to coal reservoir permeability. Additionally, a numerical simulation of CBM extraction was carried out to show the potential of the proposed approach in the application of tackling practical engineering problems. According to the results, not only the connectivity of fractures but also variations of gas pressure and velocity can be displayed explicitly, which is consistent well with the actual situation.

## 1. Introduction

Coal is a kind of porous medium with many fractures formed in it after a long-term geological process. The existence of these weak structures has a great influence on the flow of coalbed methane (CBM) that can not only lead to mine hazards but also provide a substantial source of energy in both industry and households [1,2,3]. Therefore, it is of great significance to investigate the characteristics of fractures and their internal gas flow properties for both CBM exploitation and gas outburst prevention [4,5].

In terms of the investigation of flow in porous media, Darcy’s law was the earliest linear seepage model to emerge [6,7]. Since its emergence, numerous experiments and theoretical investigations on gas flow and transport properties in various porous media have been performed and reported for single-phase, multiphase, saturated, and partially saturated domains [8,9,10]. However, because of the complex structure of porous media and the limitations of considering real geological conditions, conventional experiments and theoretical achievements fail in describing the uncertainties of flow and transport properties in porous media. For example, the continuous equivalent model averages the permeability of reservoirs and overlooks the influence stemming from the discontinuity caused by fractures [11,12]. This model is similar to the “black box” and its central problem is to solve the permeability tensor. Obviously, it is not suitable for capturing some important mechanical behaviors of fractured rocks, especially when some large-sized fractures exist. Barenblatt et al. have proposed the dual medium model, though it is not suitable to describe the flow behavior of fractures [13].

With the increasingly used computer and simulation methods, many researchers have modeled porous media using a discrete fracture network (DFN), which generally contains three categories (geological-mapping-based DFN, geomechanically grown DFN, and stochastic fracture network (SFN)) [14]. A DFN has advantages in the discretization of fracture networks and preservation of the relationships between fractures and fracture sets compared to the extensively used lattice Boltzmann method (LBM) in which fractures are discretized into cells or edges [15]. Geological-mapping-based DFN illustrates fracture patterns from limited exposure of outcrops, boreholes, or tunnels [16]. Geomechanically grown DFN can reproduce natural fractures through a DFN simulator based on paleostress conditions [17]. SFN is a simple and convenient DFN model generated from statistical data of fracture network characteristics which applies various scale fractures [18]. Fracture network characteristics can be described by FGPs, which basically comprise fracture density, length, aperture, and orientation. These FGPs all have an obvious impact on the porosity and permeability of the whole fracture structure [19,20,21]. However, due to the complexity of the stress environment, formation history, and lithotype, the distribution patterns of FGPs in various areas and buried depths are different and cannot be characterized by in situ observations or some conventional laboratory approaches including nuclear magnetic resonance and mercury intrusion porosimetry. Micron computed tomography scanning technology and scanning electron microscopy have limitations in the description of large-scale fracture systems and the determination of fractures’ connectivity features [2,22]. Seismological surveys may be able to assess and image large-scale structures but the current technology can hardly detect widely-spreading medium and small fractures due to the resolution limit [23]. In contrast, SFN modelling, as a probabilistic realization of a fracture network based on the theory of random processes like the Monte Carlo method [24,25], provides an efficient way to generate fracture networks containing differently distributed FGPs derived from field observations and measurements [26,27,28,29,30,31,32]. To date, SFN modelling has been used in many fields such as civil, mining, enhanced geothermal systems, and water resource engineering [33,34,35,36,37,38,39,40].

In the present work, even though the coal matrix is regarded as a homogeneous body with fixed porosity and permeability, SFN is used to describe the fracture system of the coal seam, which can reflect the relationships between fractures and describe the heterogeneity of the coal seam in a statistical sense. Coming to the topic of this paper, although previous researchers have made great contributions to solve engineering problems using the SFN model, few studies have directly combined it with numerical simulation software to investigate engineering problems. The intention of this paper is twofold. Firstly, a coal seam is represented by a two-dimensional SFN image. The pixels in the picture represent fractures or the matrix and the image resolution determines the accuracy of the fracture size (the higher the resolution is, the smaller the size of the fracture which can be generated). Secondly, a finite element analysis of gas flow in the coal seam is carried out in COMSOL Multiphysics 5.4 on the basis that an SFN image has been transformed to a computational domain. In this way, the temporal and spatial distribution of gas velocity and pressure is able to be reflected more intuitively, which provides a more efficient method to study the flow characteristics of CBM.

## 2. The CBM Industry with a Worldwide View

**Figure 1:**
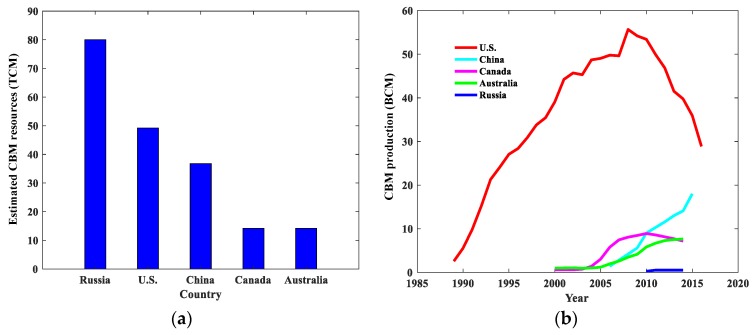
(**a**) The bar chart compares the five highest ranking countries in terms of estimated coalbed methane (CBM) resources. (**b**) The line graph shows CBM production of these five countries from 1989 to 2016, 2000 to 2015, 2000 to 2014, 2000 to 2014, and 2010 to 2014, respectively. Source: International Energy Agency https://www.iea.org/ugforum/ugd/, U.S. Energy Information Administration, Washington, DC https://www.eia.gov/dnav/ng/ng_prod_coalbed_s1_a.htm and National Development and Reform Commission http://www.ndrc.gov.cn/zcfb/zcfbghwb/201112/t20111231_585486.html.

As a kind of unconventional natural gas, CBM has been exploited in about 30 countries, and among them, the U.S. is the first and most successful country in CBM exploitation, with the largest production in the world throughout the given period shown in Figure 1b and the second largest estimated CBM resources, which are at 49.2 Tcm, as shown in Figure 1a. According to the line graph in Figure 1b, the U.S. saw a considerable increase in CBM production within the period 1989 to 2008. However, after its production peaked at 55.67 Bcm in 2008, the figure had fallen to 28.88 Bcm by 2016. This phenomenon may stem from three areas: the government provided effective policy support at first, which greatly stimulated the commercial production of CBM; later, the average daily production of single wells continued to decrease, which resulted in the economic benefits declining; and falling natural gas prices led to a sharp drop in investment. The development history of the U.S. CBM industry has important reference significance for other countries, including China.

With considerable CBM resources estimated at almost 37 Tcm, as shown in Figure 1a, China has great potential for CBM exploitation. Although it has started to exploit CBM commercially relatively late, production statistical data shows that Chinese CBM production has increased significantly from 1 Bcm in 2000 to 20 Bcm in 2015, which reflects a similar trend to the U.S.’s figures in the early years, as shown in Figure 1b. Compared to the large integrated network of CBM pipelines in the U.S., Australia, and Canada, commercial utilization of CBM in China is localized with most production coming from high-rank coals in the Ordos or Qinshui basins [41]. In terms of the geological conditions, Chinese CBM reservoirs generally reflect low permeability (under 1 × 10^−3^ µm^2^), low gas pressure, low resource abundance (under 1.3 × 10^8^ m^3^/km^2^) and great buried depth (over 600 m) compared with the U.S.’s CBM reservoir conditions (permeability over 2 × 10^−3^ µm^2^, resource abundance over 2 × 10^8^ m^3^/km^2^, and buried depth under 500 m) [42]. With these challenges faced with the development of the Chinese CBM industry, it is of great importance to have a good understanding of CBM occurrence conditions (especially pores and fractures within reservoirs, which have fundamental impacts on gas transport).

## 3. SFN Reconstruction and Processing

In this section, the basic theory for SFN generation, SFN image processing, and the techniques adopted for transforming an SFN image into a computational domain are presented.

### 3.1. Basic Theory and Method of SFN Reconstruction

The Monte Carlo method, which is known as a statistical simulation method, is based on the large number theorem and the central limit theorem. The basic idea is that when the problem is the probability of a random event, the probability of the random event is estimated by the frequency of the occurrence of this event by some "experimental" method or some digital features of the random variable. The main means of the Monte Carlo method is to use random numbers to carry out statistical tests and produce random numbers that follow a certain distribution function, which basically contains two steps:

(1) The linear congruence method is used to generate uniformly distributed random numbers in [0,1] interval [22], i.e.,

(1) $$\left\{ \begin{array}{l} x_{i+1}=\left(ax_{i}+c\right)mod\left(m\right)\\ \xi_{i+1}=\frac{x_{i+1}}{m} \end{array}\right.$$

where *x_i+1_* is a random variable corresponding to a random number *ξ_i+1_*; *a* is a multiplier; *c* is the increment; *m* is a modulus; *mod(m)* represents the remainder of the modulus; the subscript *i* is an integer; and the initial value is zero.

(2) The obtained uniformly distributed random numbers are used to generate other random numbers that are subject to different distributions based on statistical data (averages and standard variances of FGPs). 

Taking normal distribution as an example, the probability density function is expressed as

(2) $$f(x)=\frac{1}{\sqrt{2\pi }\sigma }e^{-\frac{{\left(x-\mu \right)}^{2}}{2\sigma^{2}}},-\infty <x<+\infty$$

Furthermore, the probability distribution function can be derived as

(3) $$F(x)=\int_{-\infty }^{x}f(t)dt=\frac{1}{\sqrt{2\pi }\sigma }\int_{-\infty }^{x}e^{\frac{1}{2}}(\frac{t-t^{-2}}{\sigma })dt$$

The random number of normal distribution can be obtained as

(4) $$x=\mu_{x}+\sigma_{x}\sqrt{-2\ln\xi }\cos\left(2\pi \xi \right)$$

where *x* is a random number which is subject to normal distribution; and *ξ* is a random number of uniform distribution in the [0,1] interval.

Four distribution functions in Table 1 and four FGPs of SFN (density (*ρ*), fracture length (*l*), fracture aperture (*d*), and fracture direction (*θ*)) have been investigated in this paper. A single fracture is represented by a straight line in SFN. The center coordinates of a fracture are (x_0_, y_0_) and center points of all fractures are uniformly distributed in an SFN. By using Equation (5), the starting point and endpoint coordinates of a fracture can be obtained. In addition, the number of fractures is determined by fracture density, and the fracture orientation is defined by the angle from the X-axis rotated along counter clockwise to the fracture. In this way, SFNs can be reconstructed.

(5) $$\left\{ \begin{array}{l} x=x_{0}\pm (\frac{l}{2})\cos\theta \\ y=y_{0}\pm (\frac{l}{2})\sin\theta \end{array}\right.$$

### 3.2. SFN Image Processing

**Figure 2:**
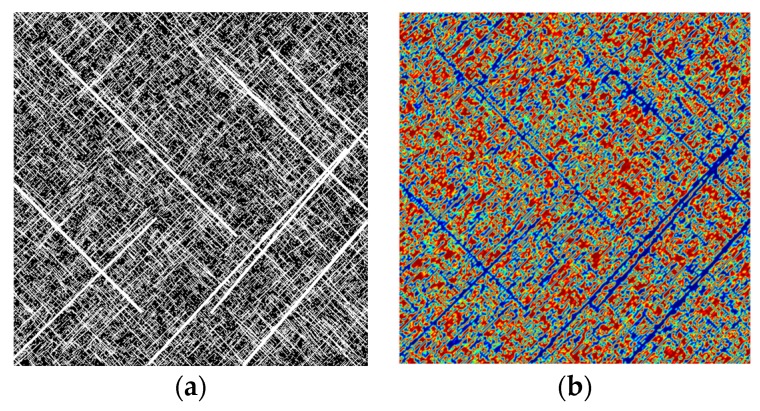
The process of transforming a stochastic fracture network (SFN) image to a computational domain: (**a**) a stochastic fracture image generated and processed in MATLAB; (**b**) the same image which has been imported into COMSOL through the image function.

For the sake of simplicity and to show the efficient use of such an SNF model in a numerical simulation, the simulations were restricted to two-dimensional images in the present study. A series of images representing fracture structures were obtained and processed through MATLAB 2016b for reconstructing the coal reservoir. Figure 2a represents an SFN which has been processed for frame removing, grey processing, binarization, and color reversion. The white regions depict fractures and the black regions depict the coal matrix. Figure 2b processed by image function defines a continuous computational domain for CBM flow simulation in COMSOL. Similarly, based on these steps, other SFN images were processed to obtain different computational domains.

### 3.3. Image function

The image function makes it possible to import an image to COMSOL and map the image’s RGB data to a scalar (single channel) function output value. By default the function’s output uses the mapping (R+G+B)/3. An image is defined on a two-dimensional domain, and we typically describe the image function using spatial coordinates: *im(x,y)*. According to Section 3.2, we made all the images binarized, so *im(x,y)* = 0 or 1. If the area on the image represents the coal matrix (the red area in Figure 2b), *im(x,y)* = 0, and where *im(x, y)* = 1 this represents fractures (the blue area in Figure 2b). Therefore, the porosity and permeability of an SFN can be divided into two parts by the image function using

(6) $$\varphi =\left(\varphi_{f}-\varphi_{m}\right)\times im(x,y)+\varphi_{m}$$

(7) $$K=\left(K_{f}-K_{m}\right)\times im(x,y)+K_{m}$$

where *φ* is the porosity of the SFN; *φ_m_* is the porosity of the coal matrix; *φ_f_* is the fracture porosity; and *K* is the permeability of the SFN. *K_m_* is the permeability of the coal matrix and *K_f_* is the permeability of fracture.

## 4. Numerical Simulation of Gas Flow in the Coal Seam

The basic assumptions, computational geometry, governing equations, and numerical techniques adopted for investigating the influence of different FGPs on the permeability of the SFN are presented in this section.

### 4.1. Basic Assumptions

In this study, the following basic assumptions were made:

(1) The coal seam is represented by an SFN and treated as a dual-porosity reservoir that is composed of fractures and the coal matrix.
(2) FGPs consist of density, length, aperture, and orientation.
(3) The flow in the coal seam is a single phase and saturated Darcy flow.
(4) Gas absorption is described by the Langmuir law.
(5) Coupling effects of multiple physical fields are ignored.

### 4.2. Fracture Flow Model

**Figure 3:**
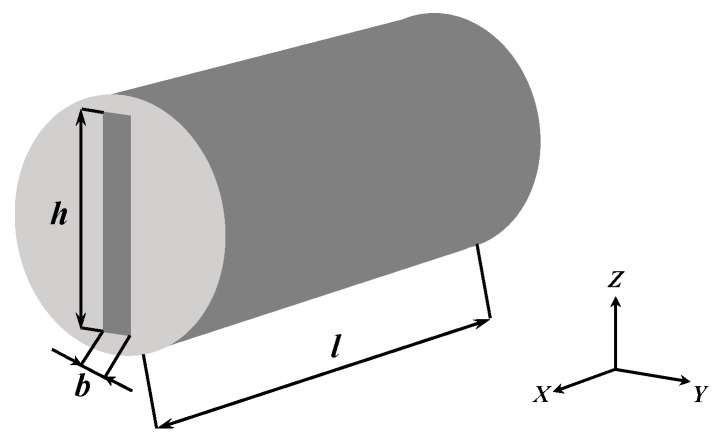
Diagram of a 3D fracture in coal.

Numerous investigations including theoretical analyses and numerical modeling about the flow behavior in fractured rocks have been conducted with various rocks. Researchers have normally assumed laminar flow in a single fracture with two fracture surfaces. According to the Navier-Stokes equation, the average flow rate through a plane void can be calculated. It has been found that flow transmissivity is proportional to the cube of the fracture aperture, which also known as the “cubic flow equation” [43], i.e.,

(8) $$q=-\frac{b^{3}}{12\mu }\frac{dP}{dx}$$

where *q* is the flow rate of a fracture at a unit height in the Z direction (Figure 3); *b* is the fracture aperture; and *μ* is the fluid dynamic viscosity.

As shown in Figure 3, when the height of the fracture is *h*, the flow rate *Q* of the total rock cross section can be described as

(9) $$Q=hq=-\frac{A\varphi_{f}b^{2}}{12\mu }\frac{dP}{dx}=-\frac{K_{f}A}{\mu }\frac{dP}{dx}$$

where *A* is the area of the rock cross section.

Then the permeability of the fracture becomes:

(10) $$K_{f}=\frac{\varphi_{f}b^{2}}{12}$$

In this work, *b* is the average value of the fracture aperture in the SFN and *φ_f_* is defined by the ratio of pixels representing fractures to total pixels.

### 4.3. Governing Equations and Boundary Conditions

Based on the basic assumptions of the SFN model and ignoring gas adsorption, the continuity equation of gas flow in the coal seam can be expressed as

(11) $$\left\{ \begin{array}{l} \nabla \cdot \left(\rho_{g}V\right)=0\\ V=-\frac{K}{\mu }\nabla P\\ \rho_{g}=\frac{M_{g}P}{RT} \end{array}\right.$$

where *ρ_g_* is the gas density; *V* is the gas velocity; *µ* is the dynamic viscosity of gas; *P* is the gas pressure; *M_g_* is molar mass of gas [kg/mol]; *R* is the gas constant [J/(mol·K)]; and *T* is the temperature of the coal seam.

By substituting Equations (7) and (10) into Equation (11), the gas flow equation can be derived as:

(12) $$\nabla \cdot \left[\frac{M_{g}P}{RT}\left(-\frac{K_{m}\left(\frac{\varphi_{f}b^{2}}{12K_{m}}-1\right)im(x,y)+K_{m}}{\mu }\nabla P\right)\right]=0$$

**Figure 4:**
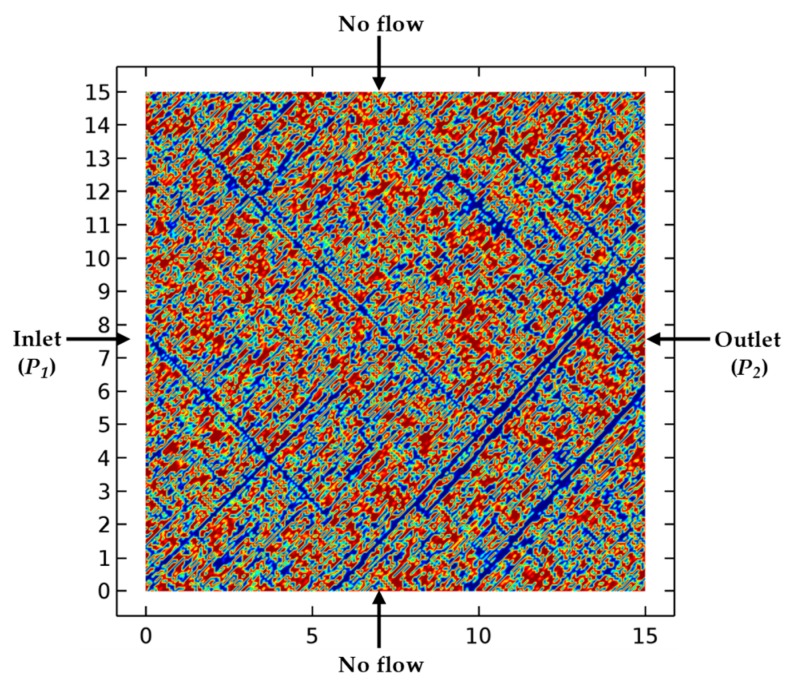
The representative computational geometries used for the numerical study. All dimensions are in meters.

The geometry of the fracture network and boundary conditions are shown in Figure 4. This geometry was imported from the processed image, with dimensions along the X and Y axes being both 15 m. The gas flow is pressure driven with a constant pressure gradient maintained from the inlet to the outlet.

### 4.4. Numerical Simulation Results

**Figure 5:**
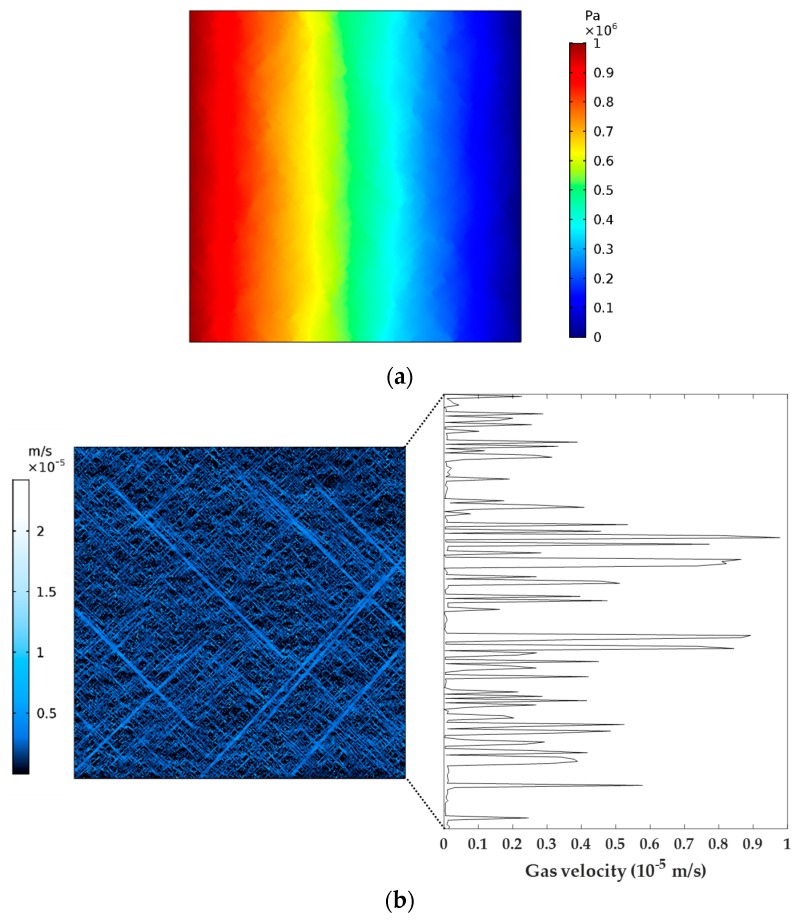
Numerical simulation results: (**a**) the spatial distribution features of gas pressure in the SFN; (**b**) the distribution of the gas velocity in the whole network attached with gas velocity at the outlet of the SFN.

Based on the boundary conditions (Figure 4), combined with the parameters in Table 2, the distribution features of gas pressure and velocity in the SFN were obtained through steady flow computation in COMSOL, as shown in Figure 5. The gas pressure was seen to gradually decrease from the inlet to the outlet, and the gas velocity in the fractures was much greater than that in the matrix. Subsequently, the average value of the gas velocity at the outlet of the fracture network was able to be obtained by integral and averaging (Figure 5b).

## 5. Parametric Study

In this section, the purpose was to compare the permeability of different fracture networks without obtaining their values. According to Darcy’s law of gas seepage in porous media (Equation (14)), the permeability (*K*) is proportional to gas velocity (*V*) when all the other parameters are fixed. Hence, the permeability was able to be compared by the variation of mean gas velocities obtained from velocity curves like Figure 5b.

(13) $$K=\frac{2QP_{\alpha }\mu L}{A(P_{1}^{2}-P_{2}^{2})}=\frac{2VP_{\alpha }\mu L}{P_{1}^{2}-P_{2}^{2}}$$

where *P_α_* is the standard atmospheric pressure and *L* represents the distance between the gas inlet and the outlet in the direction of the gas pressure gradient.

To investigate the influences of FGPs on the permeability of the coal seam, a parametric study was performed by varying every parameter and calculating the corresponding mean gas velocity at the outlet of the SFN. The reference values of the FGPs are listed in Table 3. In this part, several groups of SFN images are described in each subsection and the influences of fracture density, length, aperture, and orientation on the permeability of the SFN are discussed respectively on the basis of making all the FGPs subject to normal distribution. Additionally, impacts caused by the distribution of FGPs and the combination of differently scaled fractures are studied. Every group of SFNs contains three SFN images generated with all the same FGPs, and the average value of three simulation outcomes is taken as a reference quantity.

### 5.1. Influence of Fracture Density on CBM Flow

**Figure 6:**
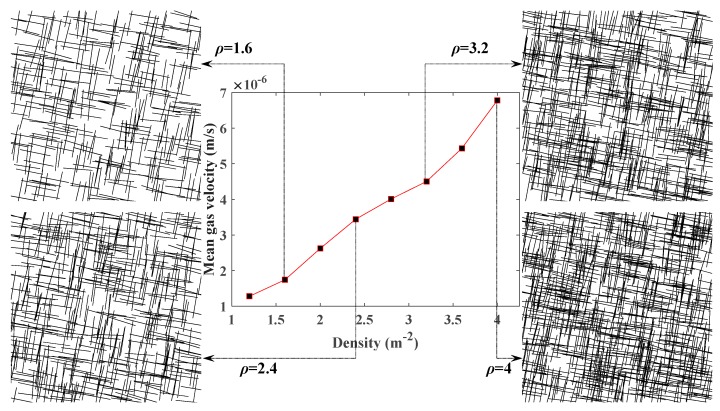
Relationship between the density of fractures and the mean velocity of gas.

To find out the influence of fracture density on the permeability of the coal seam, other FGPs have been kept the same as in Table 3. The mean gas velocity at the outlet of the SFNs generated with different densities has been compared (Figure 6). It is easy to see that the connected region increased obviously with the rising of the fracture density, which is more conducive to the flow of gas. The increasing gas velocity indicates that the permeability of the coal seam increased with greater fracture density.

### 5.2. Influence of Fracture Length on CBM Flow

**Figure 7:**
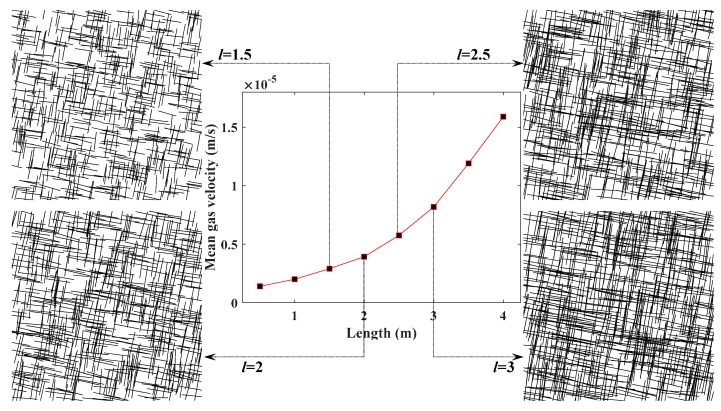
Relationship between the fracture length and the mean velocity of methane at the outlet.

To investigate the influence of fracture length on the permeability of the SFN, the mean velocity of gas at the outlet of the SFNs was compared by making the fracture length vary from 1.5 m to 3 m, as shown in Figure 7, and keeping the other FGPs the same as that in Table 3. As shown in the graph, gas velocity increased gradually with the rising of the fracture length, which indicates that longer fracture length can result in the increase of a reservoir’s permeability.

### 5.3. Influence of Fracture Aperture on CBM Flow

**Figure 8:**
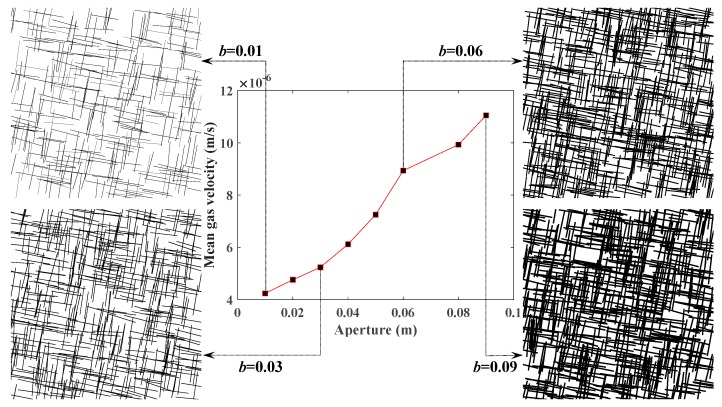
Relationship between the fracture aperture and the mean velocity of methane at the outlet.

By choosing different values of fracture aperture and the same other parameters shown in Table 3, the influence of fracture aperture on the permeability of coal seam was investigated. According to Figure 8, gas velocity rose considerably with the increase of the fracture aperture. This result from a side illustrates that the permeability of the coal seam will increase as the fracture aperture becomes larger.

As Figure 7 and Figure 8 depict, gas velocity rose by approximately 5 × 10^−6^m/s as the fracture length increased from 1.5 m to 3 m. However, gas velocity rose by almost 7 × 10^−6^m/s as the fracture aperture increased from 0.01 m to 0.09 m. Thus, it can be concluded that the fracture aperture has a more obvious effect on permeability than fracture length.

### 5.4. Influence of Fracture Orientation on CBM Flow

**Figure 9:**
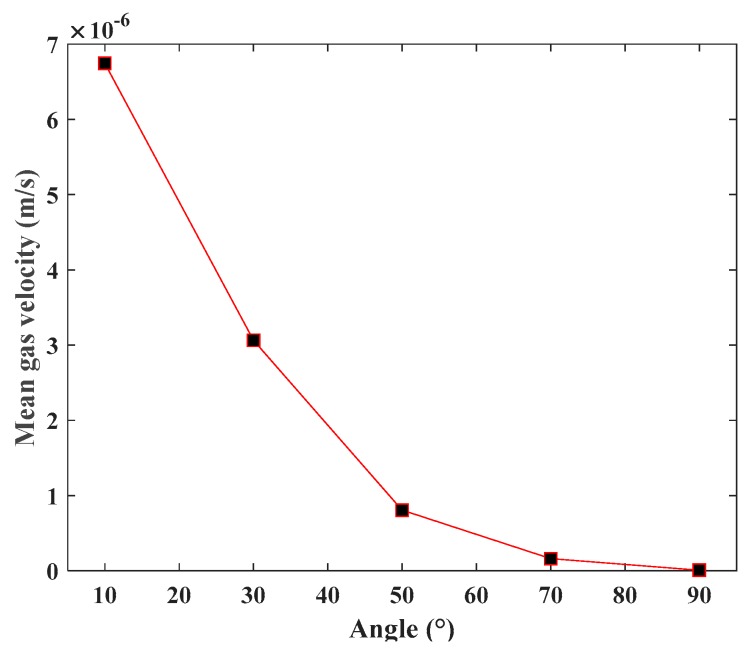
Relationship between fracture orientation and the mean velocity of methane at the outlet.

Considering the symmetry of the SFN surface, it was feasible to choose fracture direction angles of 10°, 30°, 50°, 70°, and 90° for the investigation. By making FGPs the same as that in Table 3 except for the orientation, the mean velocity of gas at the outlet of SFNs with different fracture orientations was compared (Figure 9). 

The change of fracture orientation can be seen to not affect the flow capacity of the fracture network, but it does change the direction of the gas flow. In the context of the present work, the fracture orientation means the angle between the gas flow direction and the pressure gradient. As shown in Figure 9, gas velocity decreased gradually with the increase in fracture direction angle, which illustrates that the permeability of SFN became smaller.

### 5.5. Influence of Fracture Size on CBM Flow

According to long-term field and laboratory measurements and statistics of a newly exposed coal face and collected coal samples, Fu et al. [44] proposed a comprehensive classification method of coal seam fractures through statistical analysis of the fractures’ morphological characteristics. Fracture size is divided into four grades: large fractures, middle fractures, small fractures, and micro fracture (Table 4).

**Figure 10:**
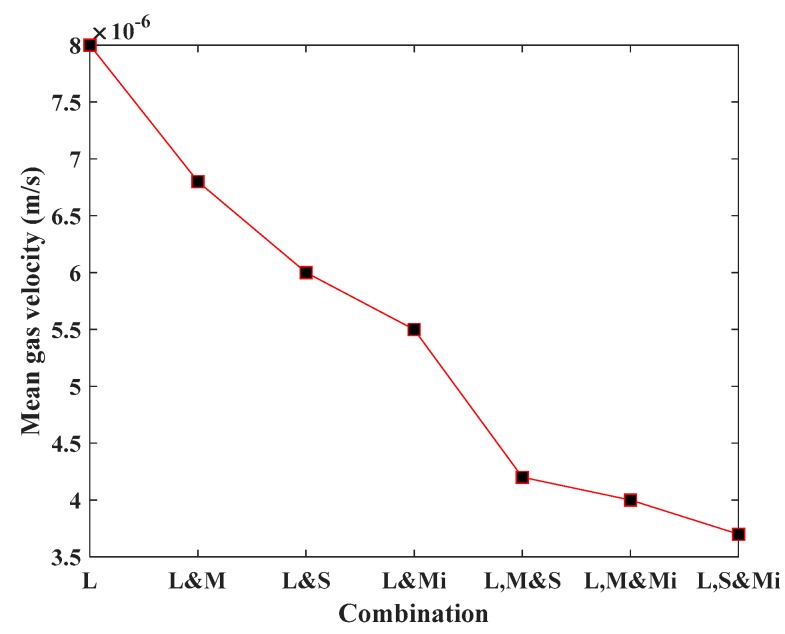
Relationship between the combination of different fractures and the mean gas velocities at the outlets of the SFNs.

In this work, seven groups of SFNs with different combinations of differently scaled fractures have been researched. The fracture densities of the SFNs were all 1.6 m^−2^ and the other FGPs were determined according to Table 4. Through numerical computation, gas velocities of each group SFN were obtained (Figure 10). It is clearly shown that the larger proportion of larger scale fractures in the SFN corresponded to greater gas flow velocity, which indicates that large scale fractures make a dominating contribution to the permeability of the coal seam.

### 5.6. Influence of Distribution Patterns of FGPs on CBM Flow

**Figure 11:**
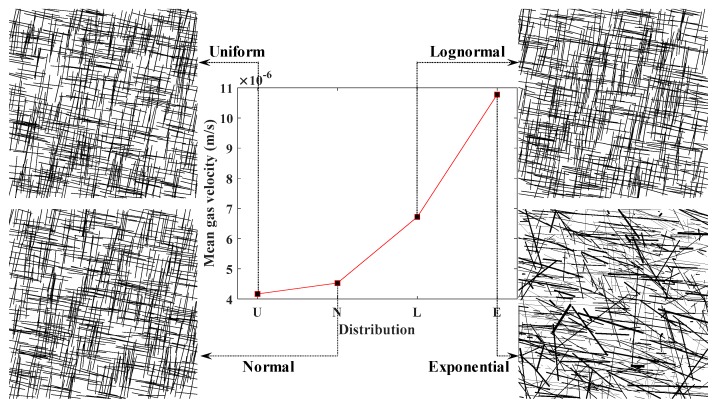
Comparison of gas velocities when FGPs are differently distributed.

The distribution patterns of FGPs are reflected by distribution functions. Generally, distribution patterns of different FGPs are not the same in the real case. In this paper, uniform distribution, normal distribution, lognormal distribution, and exponential distribution were selected to study the influence of different distribution patterns of FGPs on the permeability of the SFN, which correspond to four groups of SFNs, with all the FGPs being set to follow one distribution function (Figure 11). The fracture density SFNs were all 3.2 m^−2^, with the other FGPs kept the same as that in Table 3.

The gas velocity curve (Figure 11) shows that when the FGPs were exponentially distributed, the gas velocity was the largest, with lognormal distribution followed by normal distribution, and with gas velocity being the smallest when the value of the FGPs was uniformly distributed.

**Figure 12:**
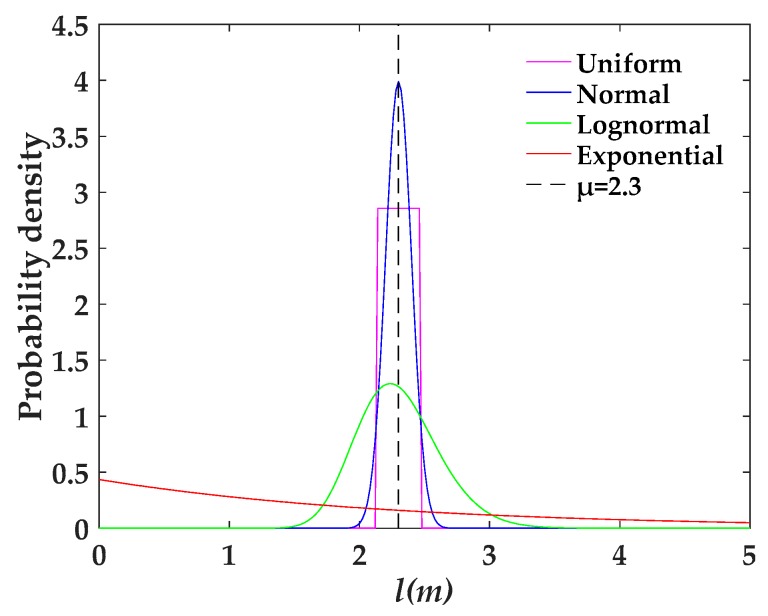
The probability density distributions of fracture length with μ = 2.3 m; σ = 0.1 m.

In order to illustrate this result, a comparison of four probability density plots with the same mean and variance of fracture length is taken as an example. As shown in Figure 12, normal distribution is symmetric around the point *x* = *μ*, which is at the same time the mean of the distribution. Lognormal distribution is a positive skew distribution with its peak shifted to the left and a long tail to the right side of the mean. When the standard deviation is small, lognormal distribution is shown to be very close to normal distribution in the short term; however, lognormal distribution has more values of fracture length distributed upward in the long run. For uniform distribution, the probability density is constant within two boundaries and the value range of the fracture length is smaller compared with the normal distribution. Relatively speaking, the probability density change of the exponentially distributed fracture length is small, and the variable values are more widely distributed than that in the lognormal distribution. Therefore, combined with the conclusion of the previous subsection, the result of Figure 11 can be well supported.

## 6. CBM Extraction Simulation Based on SFN Modeling

**Figure 13:**
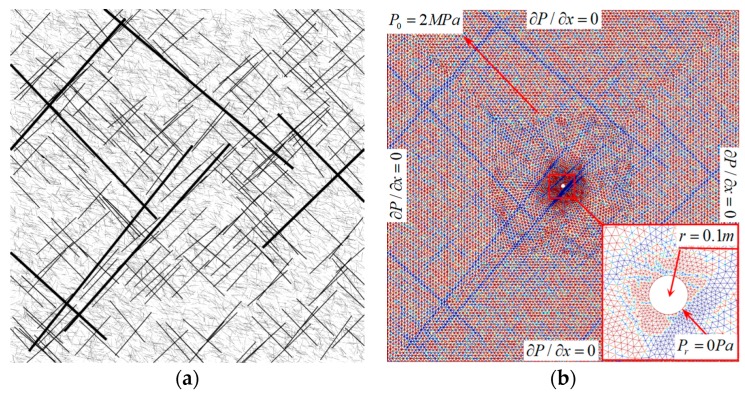
(**a**) The SFN generated for the gas extraction simulation; (**b**) the same image which has been imported into COMSOL and for which a mesh containing 28,676 elements has been generated in the computational domain. Boundary conditions and initial conditions have also been indicated in the figure.

In order to test the application in solving practical engineering problems through the proposed approach, an SFN image with four scales of fractures was generated to represent the coal seam (Figure 13a). Based on Section 4, a CBM extraction numerical simulation with consideration of gas adsorption and desorption was carried out in this part. Boundary conditions and initial conditions adopted in the simulation are shown in Figure 13b.

In practical engineering applications, the data of FGPs are collected by field and laboratory measurements and then the probability density functions are determined according to the fitting of the data. For the sake of simplicity, we determined that the fracture lengths all obey a lognormal distribution; the direction angles of the large and middle fractures obey a lognormal distribution; the direction angles of the small and micro fractures obey an exponential distribution; and fracture apertures at all scales follow a normal distribution. The specific values of the FGPs are shown in Table 5. The numerical simulation parameters in COMSOL have been derived from Table 6.

### 6.1. Governing Equations and Parameters

CBM content in the reservoir consisted of absorbed gas and free gas, which is defined as [45]

(14) $$m_{g}=\frac{M_{g}P}{RT}\varphi_{f}+\rho_{\mathrm{gs}}\rho_{s}\frac{V_{L}P}{P_{L}+P}$$

where *ρ_gs_* is the density of the gas under standard conditions; *ρ_s_* is the coal skeleton density; *V_L_* is the Langmuir volume constant; and *P_L_* is the Langmuir pressure constant.

Under the influence of the gas concentration and pressure gradient, the gas in the matrix is shown to migrate into fractures. On the basis of mass conservation, Equation (15) is able to be obtained, i.e.,

(15) $$\frac{\partial m_{g}}{\partial t}+\nabla \cdot \left(\rho_{g}V\right)=0$$

Substituting Equations (7), (10), and (14) into Equation (15), the gas migration equation in the coal seam can be written as

(16) $$\begin{array}{l} \frac{\partial }{\partial t}\left\{\frac{V_{L}P}{P_{L}+P}\rho_{s}\rho_{gs}\right\}+\frac{\partial }{\partial t}\left(\varphi_{f}\frac{M_{g}}{RT}P\right)\\ +\nabla \cdot \left(-\frac{M_{g}\left(\frac{\varphi_{f}b^{2}}{12K_{m}}-1\right)im(x,y)K_{m}+M_{g}K_{m}}{\mu RT}P\nabla P\right)=0 \end{array}$$

### 6.2. Simulation Results Analysis

**Figure 14:**
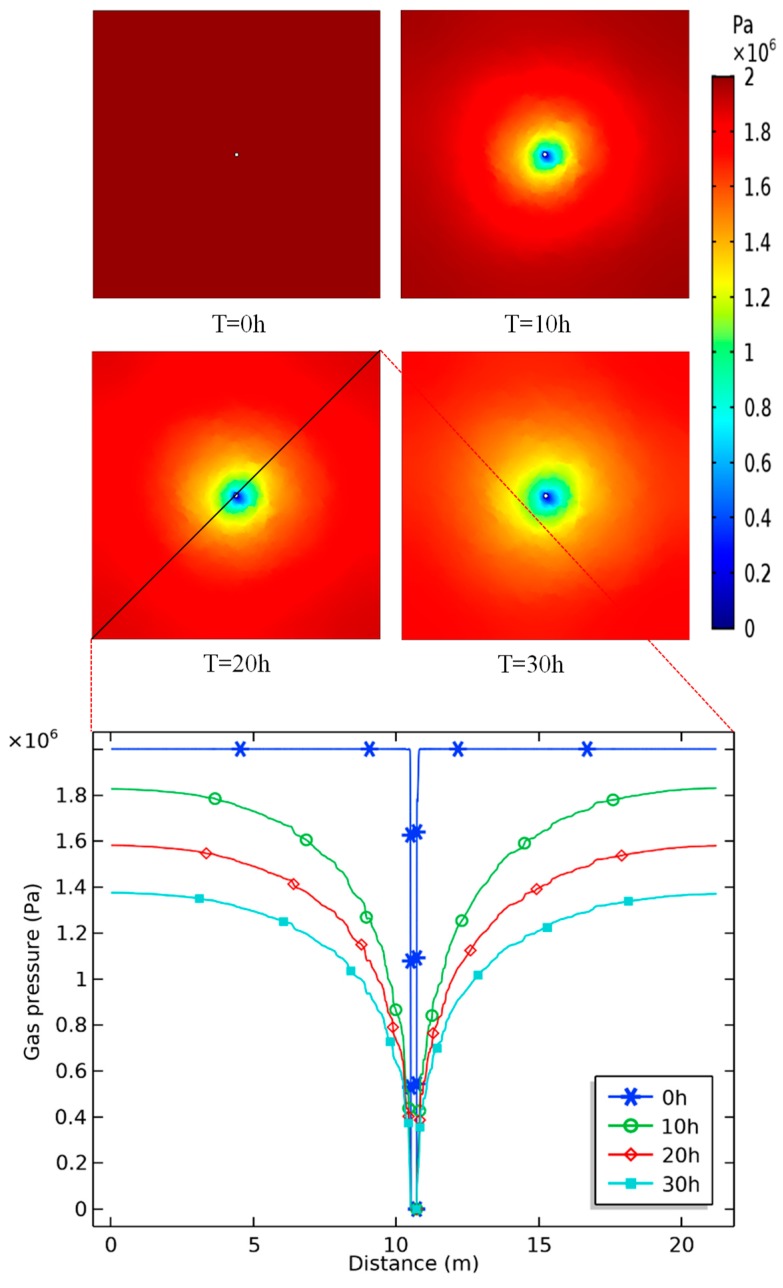
Gas pressure distribution around the borehole at different times.

**Figure 15:**
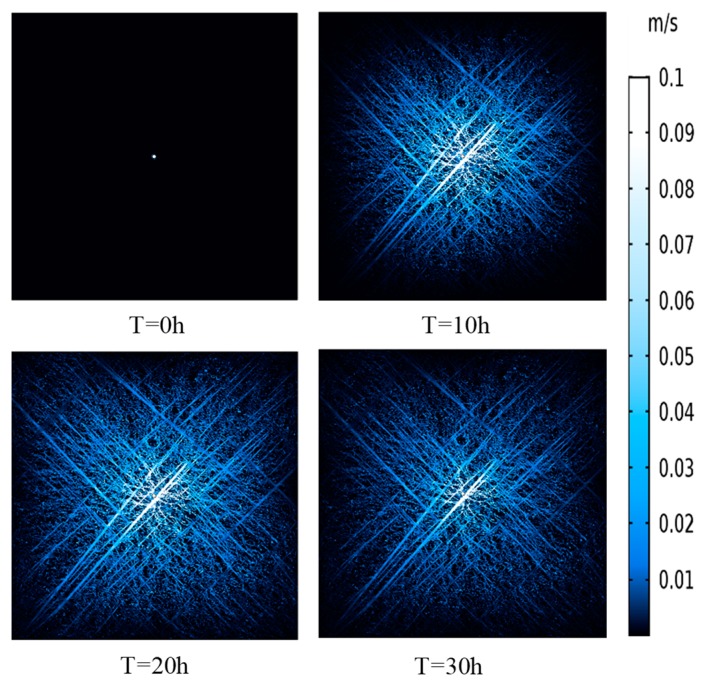
Gas velocity distribution around the borehole at different times.

Figure 14 gives information about the spatial and temporal distributions of gas pressure in the coal seam at four points at the times 0, 10, 20, and 30 h. It is apparent that the gas pressure around the borehole gradually decreased with time. The pressure decreased quickly near the borehole and the decrease became slower as it moved away from the borehole. This phenomenon resulted in the pressure drop funnel forming around the borehole, which can also be observed in the line graph. Figure 15 illustrates the gas velocity distribution in the coalbed at different times. It is noticeable that the gas velocity in fractures was much larger than that in matrix. Gas velocity increased from T = 0 h to T = 10 h and then decreased with the gas pressure becoming small. Additionally, the area where the gas velocity changed obviously got larger first and then became smaller. The simulation results show the characteristics of CBM flow during the process of gas extraction, which indicates that SFN images combined with finite element analysis have great potential in the application of tackling engineering problems.

## 7. Conclusions

Traditionally, pore-fracture scale simulations are conducted using the lattice Boltzmann method. However, in this work a relatively simple technique to show CBM migration through finite element analysis, which is based on 2D SFN modeling and image function, has been proposed, in which the dual-porosity medium coalbed is represented by an SFN image which can be generated by a self-built program in MATLAB. Influences of different FGPs and their distributions on the permeability of SFN were analyzed and a CBM extraction simulation in COMSOL was carried out. Although some limits, such as generating SFNs without taking other FGPs like tortuosity and roughness into consideration, difficulty in combining large scale fractures with nanoscale pores, and the absence of multi-field coupling effects analysis, still exist, the proposed method provides an efficient way to research CBM flow properties in the coal seam, which has great potential not only for gas-induced hazard prevention but also CBM industry development. According to the present study, the following conclusions can be drawn: 

(1) Based on the Monte Carlo method, SFNs with different FGPs were able to be generated, with the simulation results showing that the permeability of SFN increases with larger values of density, length, and aperture, and smaller values of the angle between the fractures and gas pressure gradient. The fracture aperture has a larger influence on permeability than fracture length according to the variation range of gas velocity and the value of FGPs (Figure 7 and Figure 8).

(2) The contribution order of different scales of fractures to the coal reservoir permeability from large to small is: large-size fractures, middle-size fractures, small-size fractures, and micro fractures, which also confirms the first conclusion because larger fracture size is shown to correspond to larger trace length and aperture.

(3) The impacts on reservoir permeability of FGP distribution were examined. On the condition that the values of all FGPs are kept the same, permeability ranking of SFNs from large to small is SFN with exponentially distributed FGPs, with lognormally distributed FGPs, with normally distributed FGPs, and with uniformly distributed FGPs.

(4) The gas extraction simulation can reflect CBM flow properties at each stage of the entire extraction process, including the temporal and spatial variations of gas velocity and pressure, the differences of gas velocity and pressure between fractures and coal matrix, and the gradually formed gas pressure drop cone.

## Figures and Tables

**Table 1:** The probability density functions and corresponding random variables used in this work.

Distribution	Probability Density Function	Random Variable
Uniform	$f(x)=\frac{1}{b-a}(a\leq x\leq b)$	$x_{r}=(b-a)\xi +a$
Normal	$f(x)=\frac{1}{\sqrt{2\pi }\sigma }e^{-\frac{{\left(x-\mu \right)}^{2}}{2\sigma^{2}}}$	$x_{r}=\sqrt{\frac{12}{n}}\left(\sum_{i=1}^{n}\xi_{i}-\frac{n}{2}\right)\sigma +\mu$
Exponential	$f(x)=\frac{1}{\mu }e^{-(x/\mu )}(x>0)$	$x_{r}=-\mu \ln(1-\xi )$
Lognormal	$f(x)=\frac{1}{\sqrt{2\pi }\sigma x}e^{-\frac{1}{2}{\left(\frac{\ln x-\mu }{\sigma }\right)}^{2}}(x>0)$	$x_{r}=e^{\sqrt{\frac{12}{n}}\left(\sum_{i=1}^{n}\xi_{i}-\frac{n}{2}\right)\sigma +\mu }$

**Table 2:** Parameters used for simulation.

Parameter	Value
Gas dynamic viscosity (*µ*)	11.067 [Pa.s]
Matrix permeability (*k_m_*)	0.02 [mD]
Matrix porosity (*φ_m_*)	0.01
Gas pressure of inlet (*P_1_*)	1 [MPa]
Gas pressure of outlet (*P_2_*)	0 [MPa]
Coal seam temperature (*T*)	310 [K]

**Table 3:** Reference values of fracture geometric parameters (FGPs).

Density (m^−2^)	Orientation (°)		Length (m)		Aperture (m)	
	Mean	Standard Deviation	Mean	Standard Deviation	Mean	Standard Deviation
3.2	10/100	3°	2.3	0.1	0.03	0.002

**Table 4:** Classification of coal fracture size.

Fracture Size	Fracture Aperture (mm)	Length (m)	Density (m^−1^)
Large (L)	>100	>10	1~10
Middle (M)	10~100	1~10	10~100
Small (S)	5~15	0.01~1	10~200
Micro (Mi)	<10	0.01~0.1	20~500

**Table 5:** FGPs for coalbed reconstruction.

Fractures	Density (m^−2^)	Orientation (°)		Length (m)		Aperture (m)	
		Mean	Standard Deviation	Mean	Standard Deviation	Mean	Standard Deviation
Large	0.03	45/135	3	11	0.5	0.11	0.01
Middle	1	45/135	3	2.3	0.1	0.03	0.002
Small	30	45	-	0.4	0.05	0.005	0
Micro	100	45	-	0.06	0.01	0.001	0

**Table 6:** Computational parameters in simulation.

Parameter	Value
Gas density under standard conditions (*ρ_gs_*)	0.716 [kg/m^3^]
Langmuir pressure constant (*P_L_*)	3.034 [MPa]
Langmuir volume constant (*V_L_*)	0.036 [m^3^/kg]
Density of coal skeleton (*ρ_s_*)	1370 [kg/ m^3^]
